# Oral Squamous Cell Carcinoma and What We Lose During Formalin Fixation: An Evaluation of Changes in Macroscopic Resection Margins Utilizing Virtual Three-Dimensional Imaging Techniques with Analysis Based on 947 Measurements

**DOI:** 10.3390/biomedicines12122805

**Published:** 2024-12-10

**Authors:** Adam Michcik, Maksym Jopek, Rafał Pęksa, Piotr Choma, Łukasz Garbacewicz, Adam Polcyn, Barbara Wojciechowska, Tomasz Wach, Maciej Sikora, Paolo Iacoviello, Giovanni Audino, Barbara Drogoszewska

**Affiliations:** 1 Department of Maxillofacial Surgery, Medical University of Gdansk, Mariana Smoluchowskiego 17, 80-214 Gdansk, Poland; pchoma@uck.gda.pl (P.C.); lgarbacewicz@gumed.edu.pl (Ł.G.); adampolcyn@gumed.edu.pl (A.P.); barbara.wojciechowska@gumed.edu.pl (B.W.); drog@gumed.edu.pl (B.D.); 2 Laboratory of Translational Oncology, Intercollegiate Faculty of Biotechnology of the University of Gdansk and the Medical University of Gdansk, Dębinki 1, 80-211 Gdansk, Poland; maksym.jopek@gumed.edu.pl; 3 Centre of Biostatistics and Bioinformatics, Medical University of Gdansk, Dębinki 1, 80-211 Gdańsk, Poland; 4 Department of Pathomorphology, Medical University of Gdansk, Mariana Smoluchowskiego 17, 80-214 Gdansk, Poland; rafal.peksa@gumed.edu.pl; 5 Department of Maxillofacial Surgery, Medical University of Lodz, Zeromskiego 113, 90-549 Lodz, Poland; tomasz.wach@umed.lodz.pl; 6 National Medical Institute of the Ministry of Interior and Administration, Wołoska 137 Str., 02-507 Warsaw, Poland; sikora-maciej@wp.pl; 7 Department of Maxillofacial Surgery, Hospital of the Ministry of Interior, Wojska Polskiego 51, 25-375 Kielce, Poland; 8 Department of Biochemistry and Medical Chemistry, Pomeranian Medical University, Powstanców Wielkopolskich 72, 70-111 Szczecin, Poland; 9 Department on Maxillofacial and Plastic Reconstructive Surgery, E.O. Ospedali Galliera Genova, Mura delle Cappuccine 14, 16128 Genova, Italy; paolo.iacoviello@galliera.it (P.I.); audinodr.giovanni@gmail.com (G.A.)

**Keywords:** floor of the mouth cancer, formalin fixation, macroscopic resection margin, maxilla cancer, oral squamous cell carcinoma, scanning 3D, shrinkage, tongue cancer, virtual objects

## Abstract

Background: An adequate OSCC macroscopic resection margin (MRM) is essential for effective treatment. This study analyzed the effects of formalin fixation (FF) on the MRM. Material and Methods: A total of 42 patients were enrolled in this study. Tumors from the floor of the mouth (FOM; n = 23), the tongue (TC; n = 10), and the maxilla (MT; n = 9) were studied. A 3D scanner was used to create virtual models, and further analysis was conducted according to the established protocol. Results: The most significant shrinkage was observed in the TC (MRM n = 121; Med. = 1.5 mm; *p* val. = 7.05 × 10^−18^), with a maximum shrinkage of 28%. For the FOM (n = 262; Med. = 0.8 mm; *p* val. = 6.76 × 10^−18^), the greatest MRM shrinkage was 26%. In the MT group (n = 91; Med. = 0.9 mm; *p* val. = 2.69 × 10^−9^), the shrinkage was 18.7%. Among MRMs >8 mm (n = 159), FF led to 58.5% of them shrinking to ≤8 mm, resulting in a false decrease in the safe MRM (*p* val. = 1.11 × 10^−27^). Overall, the average shrinkage for all specimens was μ= 2.57 mm (*p* val. = 8.89 × 10^−10^) alongside and μ= 2.35 mm (*p* val. = 4.09 × 10^−6^) across. The tumors themselves showed minimal changes: μ= 0.69 mm (*p* val. = 9.73 × 10^−3^) alongside and μ= 0.8 mm (*p* val. = 2.52 × 10^−7^) across. Conclusion: Formalin fixation (FF) caused the shrinkage of the OSCC MRM, particularly in tongue cancers. Even after proper surgical excision in the postoperative results, the number of normal MRMs was underestimated. This should be considered when interpreting the results of surgical treatment. However, FF had a minimal impact on the overall shrinkage of the tumors themselves.

## 1. Introduction

Oral cancers are primarily classified as squamous cell carcinomas (OSCCs), which constitute a substantial group of malignant tumors observed in both developing and developed countries [1]. Despite advancements in medicine, the 5-year survival rate remains unsatisfactory [2]. Oral carcinogenesis is a multifactorial process that occurs when epithelial cells are affected by genetic alterations such as in TP53, EGFR (epidermal growth factor receptor), CDKN2A (cyclin-dependent kinase inhibitor 2a), NOTCH1 (translocation-associated notch homolog one gene), and STAT3 (signal transducer and activator of transcription 3) [3]. OSCC develops as initially asymptomatic [4], arising either de novo or from a precancerous lesion [5,6]. This type of carcinoma is primarily characterized by an endophytic growth pattern infiltrating surrounding tissues [4]. In most cases, within a relatively brief period, the infiltrative and endophytic nature of growth results in the emergence of symptoms such as pain, dysphagia, and odynophagia [7].

**Some of the prognostic factors determined by the biological characteristics of OSCC** [8,9,10,11], as well as the complex structure of their facial skeleton or frequent nodal metastases [12,13], **increase the risk of treatment failure**. The depth of invasion (DOI), lymphovascular invasion (LVI), perineural invasion (PNI), and extranodal extension (ENE) [14,15,16,17] are of significant importance and directly influence the risk of recurrence.

Emphasizing the importance of fundamental practices, such as properly performed tumor resection, is essential. The surgical margin during the excision of oral squamous cell carcinoma (OSCC) is crucial in ensuring optimal patient outcomes.

The concept of a clear, close, and involved surgical margin was introduced by the UK Royal College of Pathologists [18]. A widely accepted microscopic margin of greater than 5 mm is considered correct ([19,20] based on 18,832 cases of OSCC retrospectively analyzed, determined a margin of 4 mm to be sufficient [21]). It is important to clearly differentiate the terminology used in the existing literature, as it can sometimes be misleading. The term surgical margins is sometimes used to describe the margin of excision of macroscopically unchanged tissues [22,23]; in other cases, it is used to determine the histopathological margin of OSCC [21,24]. In this study, we utilized the term macroscopic resection margin (MRM) to avoid clinical ambiguities. The MRM refers to the area of visibly unchanged tissue surrounding resected oral squamous cell carcinoma (OSCC), extending from the outer edge of the tumor to the surgical cut line. By clarifying our terms, we minimized misunderstandings caused by the ambiguous terminology in the literature. It is widely accepted that an MRM dimension of approximately 1 cm is accurate [25]. Based on an extensive analysis conducted by Lin MC involving 18,832 patients with oral squamous cell carcinoma (OSCC) [21], we determined that a margin greater than 8 mm is the correct and safe minimum resection margin (MRM). After tumor excision, formalin fixation of the tumor is a standard procedure. The histopathological processing of the specimen typically begins 24 to 48 h post-excision. Tissue shrinkage during this period may have significant clinical implications and could lead to incorrect therapeutic decisions in subsequent management. MRM shrinkage after FF may lead to an increased number of excessively narrow margins, which in certain clinical cases could necessitate reoperation or adjuvant radiotherapy. The limited available literature that examines the changes occurring in oral squamous cell carcinoma (OSCC) after formalin fixation provides insights into the shrinkage of tissue preparations [22,26,27]. Due to the frequent absence of a detailed measurement methodology and the reliance on simple mechanical tools that only measure at specific points, we decided to conduct this study using modern 3D imaging techniques. We developed a protocol for creating virtual images of OSCC [28], which can be used in various ways, including in surgeon–pathologist communication, in mapping and describing tumors, and at oncology meetings. Creating virtual images offers us nearly unlimited possibilities for conducting and repeating measurements at countless points. The use of virtual OSCC images, compared to traditional methodologies described in the literature, represents a significant breakthrough, opening up entirely new opportunities previously unavailable.

The purpose of this study was to evaluate the alterations in the dimensions of oral squamous cell carcinoma that occur during the process of formalin fixation (FF). The shrinkage of the macroscopic resection margins (MRMs) was analyzed, and the study assessed how these changes varied depending on the location of the OSCC. It also aimed to determine the extent to which formalin fixation distorted the results. It reduced the MRM value below the assumed safe distance, which is typically preserved during surgical excision. An analysis of the MRM distribution, both >8 mm and ≤8 mm, was performed before and after FF. Additionally, lesions of all specimens (including tumors with an MRM and tumors without an MRM) were analyzed.

## 2. Materials and Methods

This analysis included 42 patients with OSCC operated on at the Department of Maxillofacial Surgery, Medical University of Gdańsk, in 2023–2024. All participants signed a written consent form. Patients with OSCC T1 to T4b located in the floor of the mouth (FOM; n = 23), tongue (TC; n = 10), and maxilla (MT; n = 9) were enrolled in the study. The age of all patients was over 18 years of age (mean age 68.4), and general diseases did not disqualify them from the study. The study cohort description is provided in Table 1.

### 2.1. Methods

**Figure 1:**
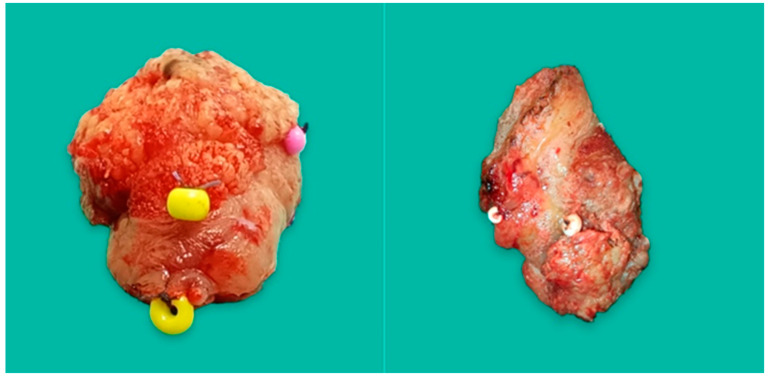
The photos show a tongue tumor with sewn-on marks to facilitate MRM determination.

Each of the patients, apart from the excision of the primary tumor, underwent a neck dissection of an appropriate scope and, in most cases, reconstruction with the use of free flaps. Patients who did not undergo en bloc resection and whose tumors did not have clear boundaries or who were damaged during resection were not eligible for this study. Only tumors where the boundary of ulceration transitioned into macroscopically unchanged tissue that was clear and reproducible to determine were examined. To minimize errors in identifying the boundary between the tumor and the MRM, each excised OSCC was marked, and an auxiliary photograph was taken. This photograph was used only if there were uncertainties during the mapping of the virtual tumor. However, in most cases, we encountered no issues after creating high-resolution virtual images of the tumors in natural colors. Furthermore, to further reduce the risk of error, each tumor was assessed four times by two trained surgeons, twice before and twice after FF. Immediately after the excision, the tumors of the oral cavity were precisely marked; markers, plastic beads of various colors, were sewn on. The markers were sewn on to the macroscopic border of the tumor transitioning into the unchanged mucosa and on the border of the surgical excision. Suturing at the tumor excision border was essential for achieving consistent measurements and facilitating the easier mapping and spatial orientation of tumors in a 3D object processing program (Figure 1).

**Figure 2:**
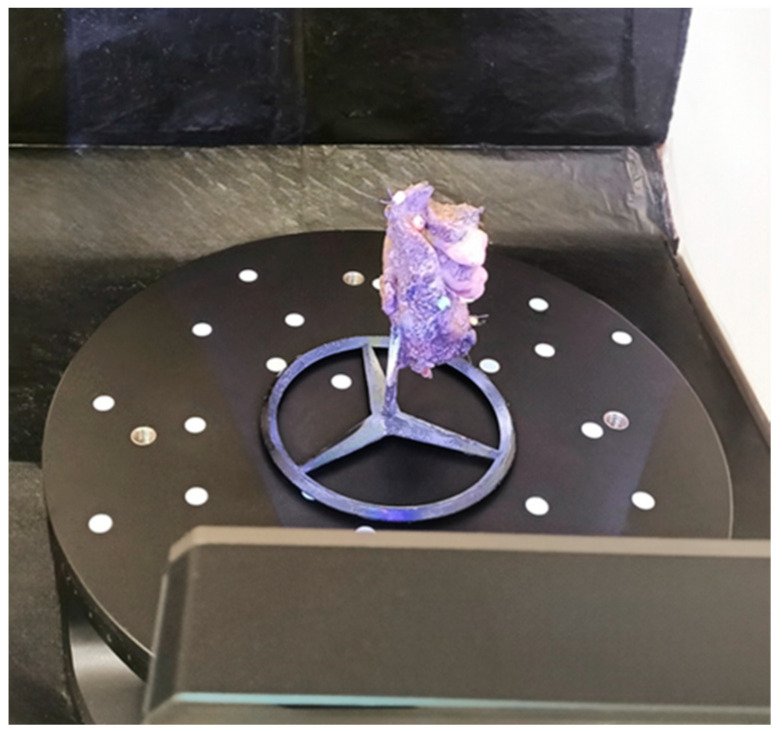
A photo of the scanner and the turntable with the constructed stand.

At least 8 beads were sewn on to each tumor. Then, the tumors were scanned in an operating room, and then they were placed in a formalin bath following a protocol. The scanning procedure consisted of four steps. First, the tumor was contrasted with a matte spray designed for Aesub Blue 3D scanners, which evaporates from an object after a short time without leaving a trace on it [29]. This allowed us to increase the accuracy of the scanned objects. Secondly, the tumor was mounted on a dedicated rotating table with a custom-designed stand (Figure 2).

**Figure 3:**
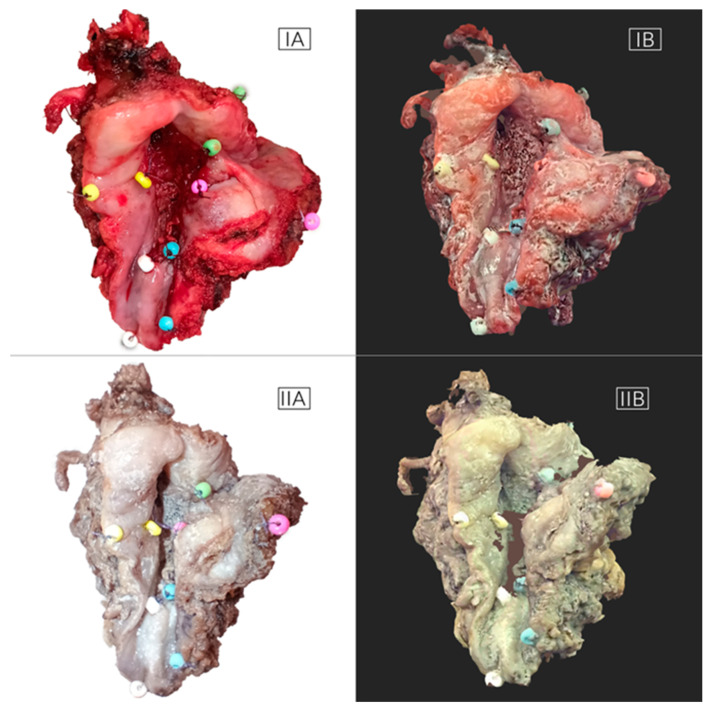
The material provides a view of the FOM tumor, highlighting the precision of the specimens obtained through the 3D scanning technique before (**IA**,**IB**) and after formalin fixation (**IIA**,**IIB**).

Its purpose was to allow the scanner to cover a broader part of the tumor. In order to further increase the accuracy of scanning, a black photographic background surrounding the scanner was used. Next, scanning was performed using a 3D scanner, the Revopoint MINI—Dual-axis Turntable Package (Shenzhen, China), adapted to scan small objects with an accuracy of 0.02 mm at a speed of 10 frames per second. The scanner was chosen for its ultra-high resolution and ambient light resistance (Figure 3).

Lastly, the obtained virtual objects in the form of STL (the triangulated representation of the surface geometry in three-dimensional space) were processed using the Revo Scan 5 program (https://global.revopoint3d.com, accessed on 2 February 2023); among other things, the environmental impurities were removed, and the surfaces of the tumor objects were sharpened. The processed 3D images were then saved as OBJ files along with the models’ textures saved as JPGs. After the tumors underwent a 24 h formalin bath in a pathology laboratory, as per the protocol, each tumor was scanned again, following the previously described procedure. This process allowed us to obtain virtual images of the oral squamous cell carcinoma (OSCC) immediately after cutting and after formalin fixation (Figure 3).

### 2.2. Measurement Methodology

The image annotation process was performed in Blender 4.1.1 open-source software (https://www.blender.org/, accessed on 25 September 2023) with a pre-installed Measure It plugin. The software was chosen for its availability, open-source status (free of charge), and easy-to-operate interface. It was originally created as a 3D modeling system for digital artists, but over the years, its uses have been expanded to movie-editing and motion capture technology. This program made it possible to apply annotations directly on the surface of 3D objects, create floating pointers with notes, and measure the object upon earlier environment scale calibration. We utilized the software capabilities to develop both objects simultaneously, and through the interaction mode, we made markings on the natural colors of the specimens.

In the first stage, a line separating the tumor from the MRM was applied to the tumor scan. Then, after the precise positioning of the second tumor scan (after FF, in relation to the first one), the scan of the first tumor was moved back in space (the line remained in the same place), and the scan of tumor no. 2 was precisely moved to the place of the first tumor. In this way, we obtained the same measurement line on both objects. Thus, we avoided errors in the subjective assessment of the determination of measurement reference points, lines separating the tumor from the MRM. Of course, we also took into account the changes in the ulcer itself after FF, but after superimposing the objects, this was also visible. Then, the surgical cut-off line was determined in parallel. Marking this line in most cases was simple and visible on the scans. At the top point of both objects, where the markers were always sewn on, we conducted the first MRM measurement at the same points, which we also verified by superimposing the objects. Then, subsequent measurements on both objects (before and after FF) took place at equal distances from the first one, which gave us the certainty of taking measurements in exactly the same places. Usually, it was every 8 or 10 mm, but it also depended on the tumor size. However, the same pair was always evaluated using the same distances.

**Figure 4:**
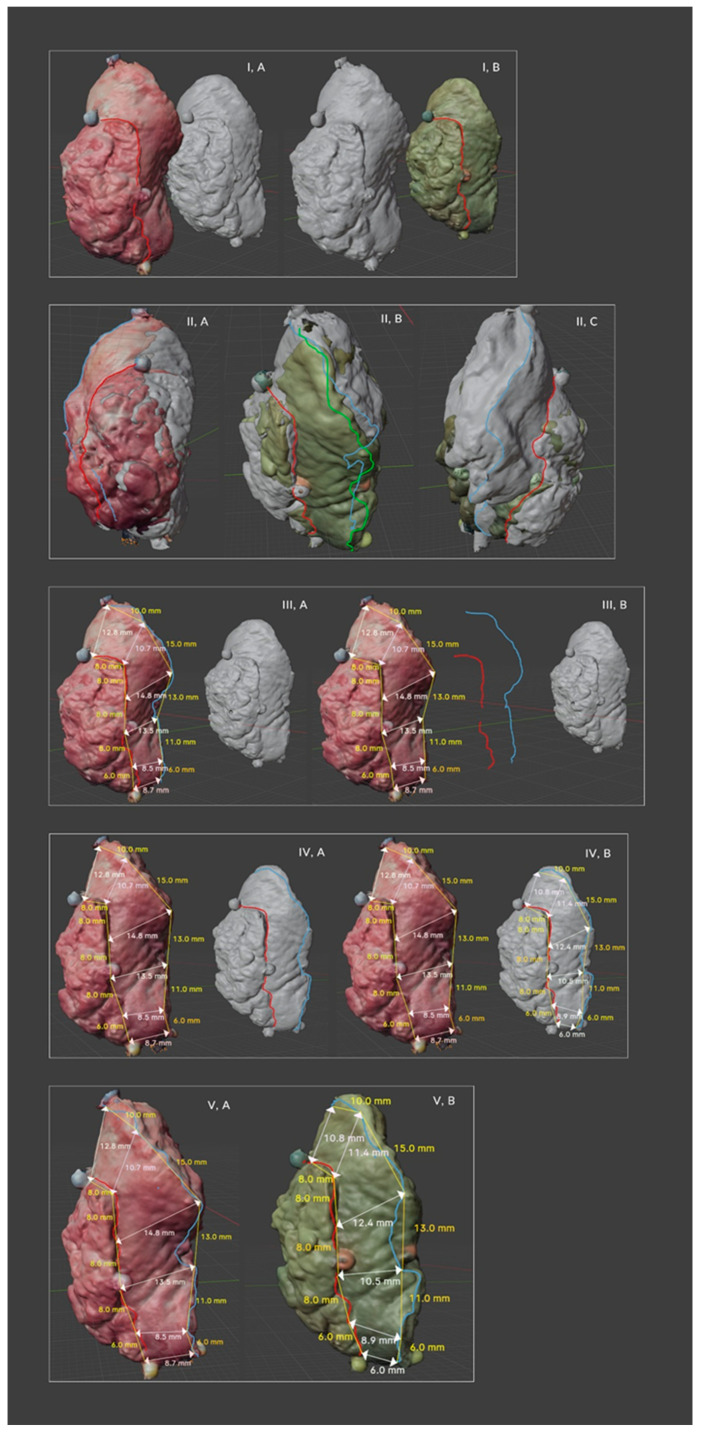
(1) Positioning objects in relation to each other (Blender 4.1.1). (**IA**)—a highlighted scan of the excised tumor (natural color); (**IB**)—an illuminated scan of the tumor after FF. (2) Overlaying objects to verify the correctness of the position necessary to perform correct measurements. (**IIA**)—an illuminated scan of the excised tumor; (**IIB**,**IIC**)—illuminated scans of the tumor after FF, viewed from two sides. (3) (**IIIA**)—taking measurements on the scan of the excised tumor; (**IIIB**)—the protrusion on the axis of the scan with measurements leaving the line separating the tumor from the MRM and the surgical excision line. (4) (**IVA**)—A scan of the excised tumor after shifting the axis of the scanned tumor after FF to exactly the same place where the measurements of the object were taken. (**IVB**)—The red line separating the tumor from the MRM remains the same and in the same place. In the verification of the surgical cut-off line, a change in the course of the surgical cut-off line was found, and a new line was drawn for the tumor/scanned object after FF. The surgical cut-off line of the first measured scan was temporarily disabled when performing MRM measurements on the tumor scan after FF. (5) (**VA**,**VB**)—A comparison of both scanned, mapped, and measured slides in natural colors, with visible differences in the MRM shrinkage after FF.

The precise use of a scanner enabled the detailed magnification of the objects to the size of individual STLs, allowing for direct drawing on the surface of virtual tumors with exceptional precision and repeatability. A total of 474 MRM measurements were taken before and after formalin fixation. An average of 11 MRM measurements were taken on each tumor before and after FF, which gave us a total of 948 measurements per investigator. As mentioned earlier, the measurements were repeated by the second surgeon, which gave a total of 1896 measurements from which the averages used for further statistical analysis were drawn (Figure 4).

Furthermore, by utilizing the specified markers and the same technology, total tumor measurements were taken in both the long and short axes using an MRM, alongside the tumor measurements without an MRM.

### 2.3. Statistics

To compare tumor margins before and after formalin fixation, we applied either a paired *t*-test (for normally distributed data) or the Wilcoxon signed-rank test (for non-normally distributed data). For comparisons involving more than two groups, we utilized the Kruskal–Wallis test, followed by post hoc pairwise comparisons with the Holm correction to adjust for multiple testings. A *p*-value of less than 0.05 was considered statistically significant for all analyses.

## 3. Results

This study revealed notable changes in the size of the excised tissue samples during formalin fixation. Both the complete samples and the MRMs themselves changed. Assuming that a macroscopic resection margin of greater than 8 mm should be preserved during the surgical procedure, it was found that formalin fixation significantly impacted the results, leading to a falsely lower count of normal MRMs.

**Figure 5:**
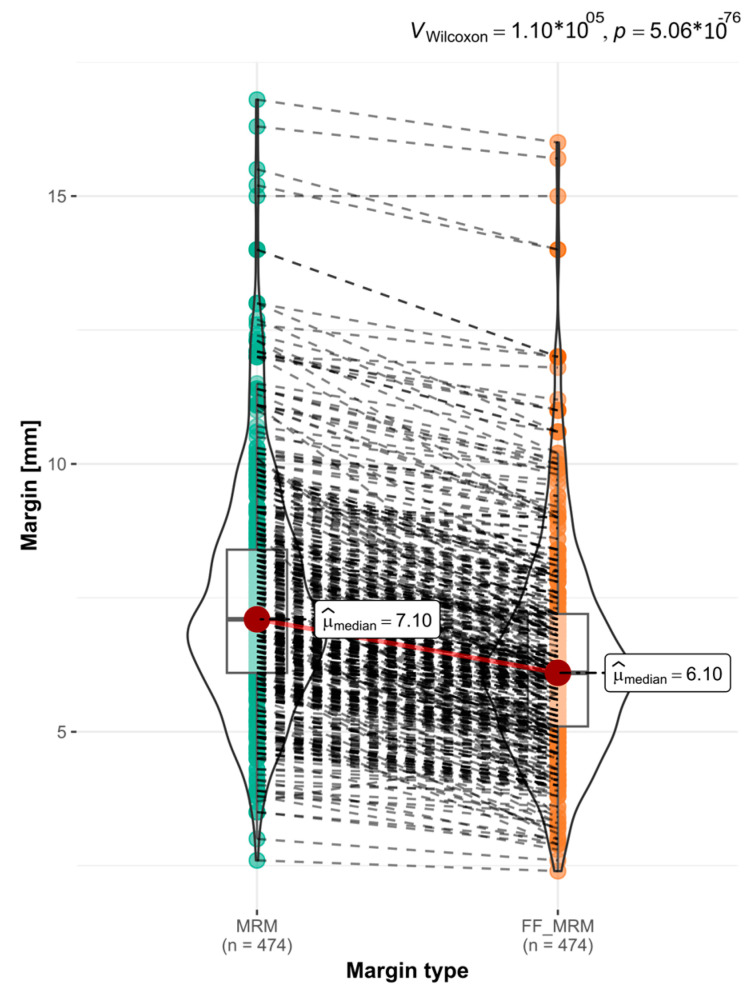
The graph presents the changes in the MRM observed after 24 h of FF. A significant reduction in the MRM is clearly evident, with a *p* val. = 5.06 × 10^−76^. The paired Wilcoxon test was employed for statistical analysis.

The analysis included 948 MRM measurements. The median MRM for the resected OSCCs was 7.1 mm, while for the MRMs after FF, Med. = 6.1 mm. The largest MRM shrinkage was 28% (*p* val.= 5.06 × 10^−76^) (Figure 5).

**Figure 6:**
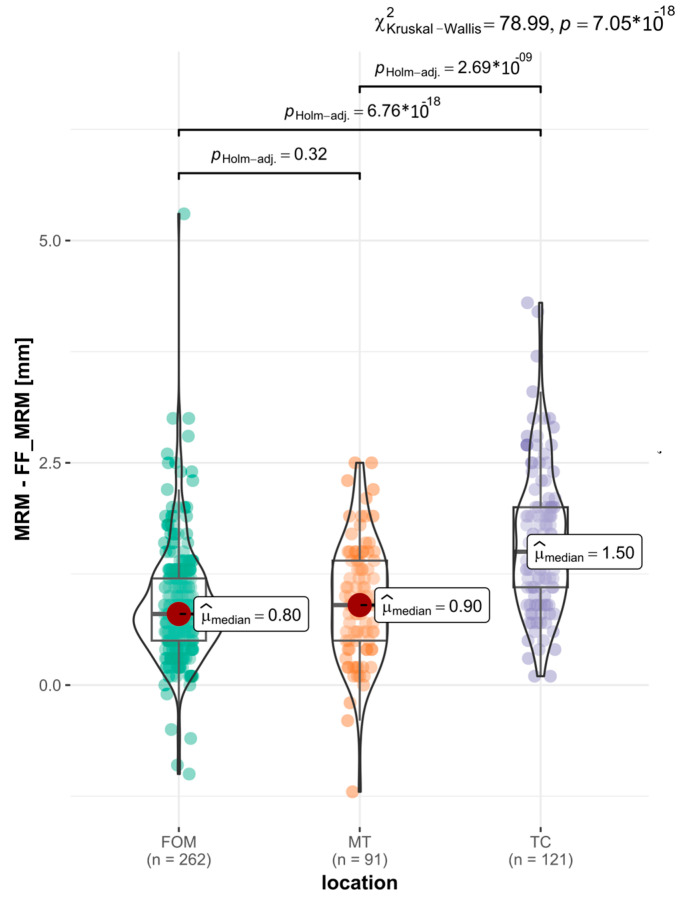
The graph illustrates the distribution of MRM changes for different OSCC locations. After applying Holm’s correction, a careful comparison of the groups revealed differences among the individual locations. A significantly greater shrinkage of the MRM after FF in the TC, with Med. = 1.5 mm, was observed. The MRM-FOM (Med. = 0.8 mm) and MRM-MT (Med. = 0.9 mm) shrinkage was at a similar level (*p* val. = 7.05 × 10^−18^).

To further investigate, we assessed whether the rate of MRM shrinkage after FF depended on the location of the OSCC. In the post hoc tests, we found statistically significant differences when comparing the TC with the FOM and the TC with the MT. Specifically, for the TC (n = 10), the MRM = 121, with a median of Med. = 1.5 mm, and the greatest MRM shrinkage was 28%. For the FOM (n = 23), MRM = 262, Med. = 0.8 mm, and the greatest MRM shrinkage was 26%. For the MT: (n = 9), MRM = 91, Med. = 0.9 mm, and the greatest MRM shrinkage was 18.7% (Figure 6).

**Figure 7:**
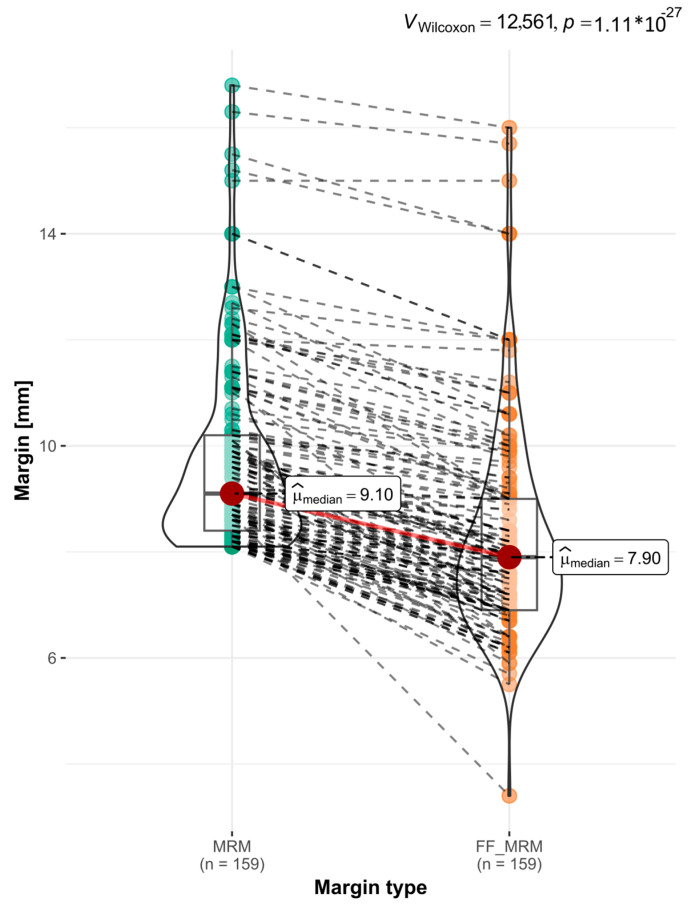
Graph of changes in distribution of safe MRMs >8 mm after FF. *p* val. = 1.11 × 10^−27^; Wilcoxon test.

Our findings indicate that the variations in the MRM size were influenced by the specific location of the tumors. Notably, the margins of the tongue tumors demonstrated the most significant reduction; for the TC, Med. = 1.5 mm., while the FOM and MT tumors shrank slightly less and at a similar level (FOM: Med. =0.8; MT: Med. = 0.9). It is important to note that we did not observe any statistically significant differences in the changes in the MRM size when accounting for the tumor size (T) or depth of invasion (DOI). We established an MRM >8 mm as both the correct and safe margin to maintain during the surgical excision of OSCC. Subsequently, we conducted an analysis of how formalin fixation affected the distribution of acceptable and unacceptable, or excessively narrow, MRMs. Among the 159 pairs of MRMs > 8 mm, formalin shrinkage reduced the dimension to below or equal to 8 mm for 93 cases (Figure 7).

In our final analysis of the excised OSCC samples, we established that FF significantly altered the MRM values, resulting in a misleading underestimation of normal results. This finding is of paramount importance from the operator’s perspective and must be considered when making therapeutic decisions.

**Figure 8:**
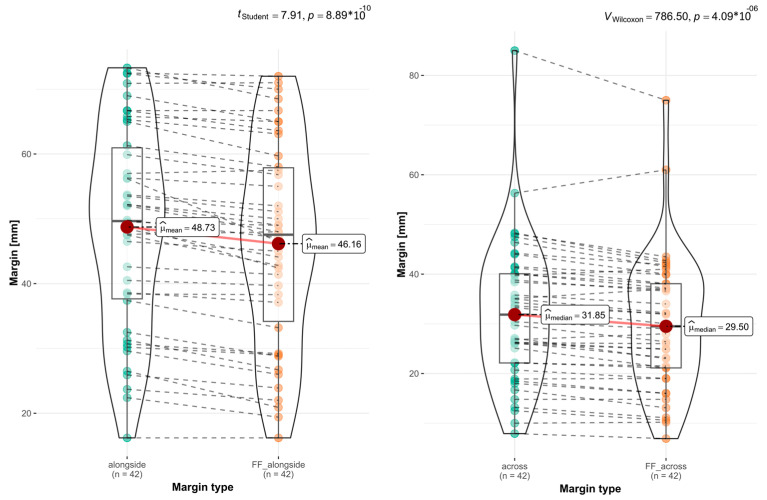
The graphs summarize the measurements of the long and short axes of the excised tumors. Notable changes in magnitude were observed following 24 h FF (*p* val. = 8.89 × 10^−10^; *p* val. = 4.09 × 10^−6^).

The last stage of the analysis included a comprehensive assessment of the OSCCs and the assessment of the tumors themselves (without an MRM). Measurements were made along the long axes of and transverse to the entire excised tumors together with the MRM and the tumors themselves (macroscopic ulcers), using the same points on the virtual STL objects according to our methodology. The evaluation of the whole preparations revealed statistically significant differences in sizes occurring during FF. The mean longitudinal shrinkage of the specimens was μ= 2.57 mm (*p* val. = 8.89 × 10^−10^), and the mean shrinkage across was μ= 2.35 mm (*p* val. = 4.09 × 10^−6^) (Figure 8).

**Figure 9:**
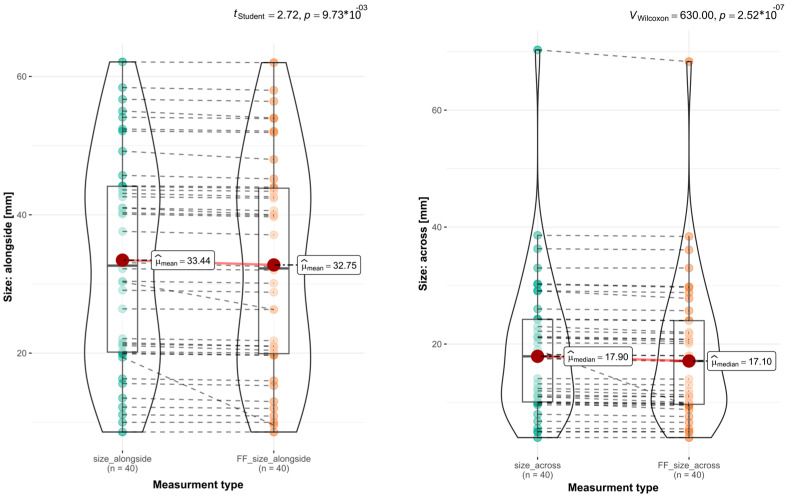
The graphs show the extent of changes in the tumor size after FF. Much smaller differences in sizes are visible before and after FF.

The final stage of our analysis involved assessing the tumors themselves, excluding the impact of MRMs. We marked the tumor area on a virtual STL object. Using this method, we could accurately determine the tumor boundaries following our measurement methodology. Tumors with unclear boundaries, such as those without obvious ulceration, were not included in this study stage. A total of 40 tumors were qualified for analysis. Our findings indicated a significantly lower shrinkage of tumor tissue during FF, measuring μ= 0.69 mm (*p* val. = 9.73 × 10^−3^) along one axis and μ= 0.8 mm (*p* val. = 2.52 × 10^−7^) across another axis (Figure 9). The results indicate that formalin fixation exerts a diminished impact on the infiltrated tissue of OSCC.

## 4. Discussion

Our analysis showed that FF significantly impacted the size of the macroscopic resection margins (*p* val. = 5.06 × 10^−76^). The use of the modern visualization techniques of 3D objects made it possible to perform many precise and repeatable measurements. In the past, we have not had the opportunity to conduct such a study, so the possibility of comparing our results with those of others available in scientific articles is minimal. For this reason, we referred to publications using a completely different, much simpler methodologies. In addition to taking measurements, creating virtual objects allowed us to archive specimens and to measure, describe, or compare them with histopathological results (currently in our clinic’s design phase). 

This work required the surgeon to leave the standard work zone and learn how to use technical software. However, the benefits were significant, especially when creating virtual communication between surgeons and pathologists [28]. An appropriate macroscopic resection margin should be maintained during OSCC resection. In the available literature, we can find information about the difficulties related to its correct determination [30]. Some authors suggest the use of optical instruments [31]. However, for technical reasons, this is only possible in selected locations. In our study, as in most of the available ones, we relied on the assessment of MRMs without optical instruments, cutting out tissue about 1 cm from the palpable tumor. OSCC excision with a safe MRM reduces the risk of recurrence [32]. The results regarding the effect of the surgical excision margin width on local recurrence risk are inconsistent. Hicks et al. demonstrated a 9% local recurrence rate in patients with 10 mm margins [33], which is significantly different from the results of Yamada et al. suggesting the preservation of 5 mm of free tissue around the tumor during surgical resection [34].

Therefore, the MRM width assumed in our study was > 8 mm, as this correct and safe margin corresponded to the available results. It should be noted, however, that the risk of local recurrence is affected not only by the surgical excision margin. Alongside this, the T stage, degree of differentiation, pN (nodal stage), LVI, PNI, DOI (ref. [35,36]), and pattern of invasion are independent risk factors for local recurrence [37]. The reducing factors are also neoadjuvant [35] and adjuvant radiation therapy [38,39], reducing local recurrence risk. When analyzing our patients’ postoperative results, we had to consider all these factors. Still, we could not have forgotten about the basics, such as maintaining the correct macroscopic resection margin when excising the tumor. In all the locations studied, we showed statistically significant differences in changes in the MRM size after FF (*p* val. = 7.05 × 10^−18^). After a thorough review of the available literature, we can conclude that our study is pioneering, and, so far, similar results have not been published. There are few studies available that assess only changes in single tumor points, and the measurements themselves were made with simple measuring instruments [26,27,40], which, for obvious reasons, were not able to assess the actual changes taking place during FF. In the papers of El-Fol H. et al. and Cheng A. et al., among others, the shrinkage of OSCC excision margins (called MRMs in our study) was compared with histopathological margins after FF [22,41]. In the opinion of the authors of this article, this was an incorrect assumption and could not properly assess the changes taking place during FF. A comparison of MRMs (macroscopic margins) with histopathological margins after FF will always show differences (assuming R0), even regardless of the effect of FF.

Through the application of virtual imaging techniques, we conducted an assessment of tumors from various angles and at multiple magnifications. A large number of measurements made it possible to group the measured MRMs into individual OSCC locations. By comparing the statistical results, we showed that the greatest shrinkage of an MRM occurs in tongue tumors (TC: n = 121 MRM; Med = 1.5 mm; *p* val. = 2.69 × 10^−9^). In the other two FOM and MT locations, we also found significant MRM shrinkage, respectively (FOM: n = 262 MRM and Med = 0.8 mm; MT: n = 91 MRM and Med. = 0.9 mm). Therefore, we had to take this fact into account when interpreting the postoperative results of the excised tumors. The results of macroscopic as well as microscopic excision margins [42] are important in terms of prognosis and affect further treatment decisions, e.g., reoperation. Maintaining an adequate MRM reduces the risk of local recurrence [31]. However, its optimal values vary from one researcher to another [21,25,30].

Based on our clinical experience and the values found in the published literature, we concluded that an MRM > 8 mm is considered safe. Upon analyzing the distribution of MRM and MRM levels after FF, we observed an increase in the number of MRMs < 8 mm post-FF. It was important to consider the observed shrinkage in nearly all measurements of the 474 MRMs, along with the changes in the quantitative distribution of safe MRMs influenced by FF, whenever interpreting the results of the postoperative examinations. Formalin fixation reduced the number of safe MRMs, despite a properly performed surgical excision procedure with originally preserved MRMs >8 mm, and falsely indicated too narrow a resection. In the assessed group with MRMs > 8 mm measurements, FF falsified the results in 58.5% of cases. This indicates that inaccurate values are reported in more than fifty percent of histopathological examinations, including MRM assessments. These incorrect values suggest an excessively narrow margin during excision, even if the actual margin is properly maintained. Due to the lack of similar studies in the available literature, we cannot compare our results with other authors. By assessing the total dimensions of resected tumors before and after FF, we obtained results indicating the shrinkage of all specimens (*p* val. = 8.89 × 10^−10^; *p* val. = 4.09 × 10^−6^). Our analysis revealed a notably lower rate of tumor shrinkage compared to tissue that appeared macroscopically unchanged (*p* val. = 9.73 × 10^−3^; *p* val. = 2.52 × 10^−7^). These findings suggest that the shrinkage of OSCC samples during formalin fixation primarily impacts MRM while having a reduced impact on the tumor itself. The infiltrative nature of tumor tissue probably causes this difference. This differential response can be attributed to the distinct structural characteristics of the tissues. The intratumoral stroma typically exhibits dense collagenization, resulting in a more compact and rigid architecture that demonstrates greater resistance to formalin-induced shrinkage compared to more pliable and loosely organized normal tissues.

Chen c et al. came to the opposite conclusion, stating that tumors shrank without margins [26]. Measurements were taken at a single point of the tumor using a caliper. This method carried a significant risk of inaccuracy due to potential variation at different points on the tumor. Consequently, we believe that our results are more reliable, especially when considering the methodology employed and the number of measurements taken. Working with virtual OSCC objects opened up valuable opportunities that had not been previously available. Our clinic intends to continue this work, particularly in conjunction with histological evaluations.

## 5. Conclusions

Formalin fixation, a widely accepted procedure for the preparation of specimens, has a significant impact on the alteration of size in excised OSCC specimens. During this process, the macroscopic resection margin (MRM) shrinks, which can lead to the misinterpretation of the final result. The assessment of excision margins in surgical oncology is of utmost importance. The erroneous interpretation of results, particularly regarding excessively narrow margins, can lead to misguided clinical decisions if the influence of factors such as FF is not taken into account. This includes a thorough reassessment of the necessity for reoperation to extend the surgical margins. In situations where it is not feasible to broaden the excision margins, the initiation of adjuvant radiotherapy must be considered. While a variety of factors influence these clinical decisions, the significance of surgical margins remains of utmost importance. Among the locations evaluated, the greatest shrinkage of the MRM was observed in tongue cancers. However, in the case of the assessment of macroscopic tumors alone, we found the smallest changes caused by formalin fixation. The use of modern 3D virtual imaging technologies allowed us to clearly demonstrate that FF changes the size of OSCC slides, mainly MRMs. This finding should be considered when interpreting and analyzing patient outcomes, as well as when making therapeutic decisions based on these data.

## Figures and Tables

**Table 1:** Characteristics of study group with 8th TNM classification used. Legends: TC—tongue cancer; FOM—floor of mouth; MT—maxilla tumor; TPS—tumor primary site; G—grading; pT—tumor size; DOI—depth of invasion; MRM—macroscopic resection margin.

Characteristic		N	%
Gender	male	30	71.4
	female	12	28.6
Age	50s	6	14.3
	60s	16	38
	70s	12	28.6
	80s	8	19
TPS	TC	10	24
	FOM	23	54.8
	MT	9	21.2
G	G1	9	21.4
	G2	26	62
	G3	7	16.6
pT	pT1	12	28.6
	pT2	12	28.6
	pT3	9	21.4
	pT4	9	21.4
DOI	≤5 mm	12	28.6
	5–10 mm	19	45.2
	>10 mm	11	26.2
MRM	TC	121	25.5
	FOM	262	55.3
	MT	91	19.2

## Data Availability

The raw data supporting the conclusions of this article will be made available by the authors on request.
